# Two-Dimensional Indium Selenide for Next-Generation Electronics

**DOI:** 10.3390/ijms27167453

**Published:** 2026-08-20

**Authors:** Donghun Lee

**Affiliations:** Department of Chemistry, Kookmin University, Seoul 02707, Republic of Korea; donghunlee@kookmin.ac.kr

**Keywords:** InSe, two-dimensional materials, Small Effective Electron Mass

## Abstract

Two-dimensional indium selenide (InSe) is a promising material for next-generation electronics, characterized by a small electron effective mass and ultrahigh room-temperature mobility. This review systematically examines the fundamental physics and emerging quantum phenomena intrinsic to InSe. It also addresses the recent discovery of sliding ferroelectricity, which breaks macroscopic spatial inversion symmetry and yields robust polarization states without conventional displacive ionic dynamics. Technological progress from mechanical exfoliation to scalable bottom-up metal–organic chemical vapor deposition is evaluated. The review also covers advanced architecture enabled by InSe, including sub-3 nm-node logic transistors and nonvolatile ferroelectric synaptic devices. Finally, key challenges involving stoichiometric control, back-end-of-line-compatible integration, and environmental instability are discussed in the context of future low-power and neuromorphic computing.

## 1. Introduction

Transition metal dichalcogenides (TMDs), such as MoS_2_ and WSe_2_, have long dominated the two-dimensional (2D) semiconductor field; however, the performance of TMD-based devices is fundamentally limited by the relatively large effective mass of their charge carriers [1,2,3]. In particular, MoS_2_ has an electron effective mass ($m_{e}^{*}$) of 0.45 *m*_0_, with *m*_0_ representing the electron rest mass, and intrinsically low room-temperature mobilities, typically ranging from 50 to 200 cm^2^ V^−1^ s^−1^ [4]. These transport limitations pose a notable barrier to realizing high-speed electronic and ballistic-transport devices. By contrast, 2D indium selenide (InSe) has a very low $m_{e}^{*}$, with typical theoretical and experimental values of 0.12 *m*_0_ and 0.14 *m*_0_, respectively [5]. Given this low mass, InSe field-effect transistors (FETs) exhibit high room-temperature electron mobilities on the order of 10^3^ cm^2^ V^−1^ s^−1^, while electrons in InSe have a high thermal velocity (*v*_th_) exceeding 1 × 10^7^ cm s^−1^ [6,7,8,9]. These transport characteristics favor quasi-ballistic transport at ultrashort channel lengths. Beyond these transport advantages, InSe also differs from TMDs in its thickness-dependent band structure. For instance, bulk MoS_2_ has an indirect bandgap that becomes direct when the material is thinned to a monolayer [10]. By contrast, InSe exhibits the opposite bandgap behavior: it retains a direct bandgap in its bulk form but undergoes a unique direct-to-indirect bandgap crossover when its thickness is reduced below a critical value, typically 5–6 nm [11,12]. In addition, InSe offers unique symmetry-based functionalities that are not readily found in conventional TMDs. Specifically, the absence of inversion symmetry in the γ and ε polytypes yields robust nonlinear optical responses, including an ultrahigh second-harmonic generation (SHG) response [13,14]. Recent research has also demonstrated stacking-sensitive polarization phenomena and ferroelectricity in InSe-based systems [15,16,17]. These unique ferroelectric properties, which arise without composition engineering or complex heterostructures, position InSe as a promising platform for advanced nonvolatile memory and neuromorphic computing architectures.

This review examines the atomic arrangement, electronic structure, synthesis, and device integration of 2D InSe. First, the principal InSe polytypes, including the ε, β, and γ phases, are introduced. The discussion then addresses InSe preparation through exfoliation-based and scalable thin-film growth methods, with particular attention to phase selectivity, stoichiometric control, and surface degradation. The InSe-based devices are subsequently introduced, with a focus on high-performance transistors, broadband optoelectronic devices, and novel memory concepts. Finally, the remaining obstacles to wafer-scale integration are identified, and the potential contributions of InSe to low-power electronics, integrated circuits, and neuromorphic computing are outlined.

## 2. Crystal Structures and Fundamental Properties

### 2.1. Crystal Structures

**Figure 1:**
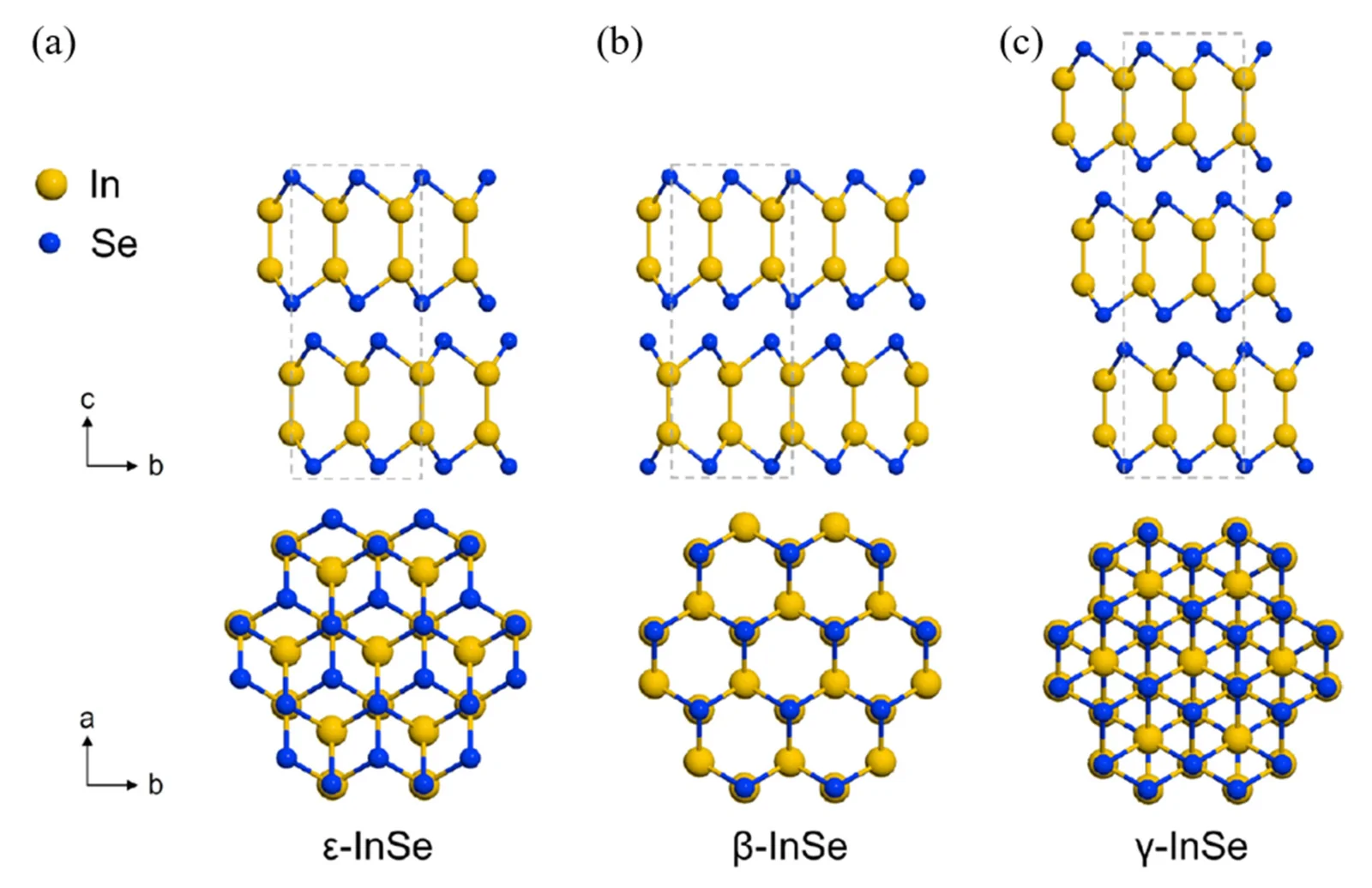
(**a**–**c**) Schematic crystal structures of (**a**) ε-InSe, (**b**) β-InSe, and (**c**) γ-InSe, with side views at the top and top views at the bottom. Yellow and blue spheres represent In and Se atoms, respectively. Reproduced with permission [17].

The fundamental building block of 2D InSe is a quadruple atomic layer comprising covalently bonded atoms arranged in an Se–In–In–Se sequence along the out-of-plane (OOP) direction [18]. Within an isolated layer, the atoms form a 2D honeycomb lattice with *D*_3h_ point-group symmetry, whereas adjacent quadruple layers are held together solely by weak van der Waals (*vdW*) interactions [19]. InSe exhibits diverse, energetically stable structural polytypes because the formation energies associated with interlayer sliding or twisting are similar across polytypes [20]. Accordingly, depending on the relative in-plane (IP) translation and rotational alignment of adjacent layers, the bulk and multilayer forms of InSe crystallize primarily as three thermodynamically stable polytypes: ε, β, and γ (Figure 1) [17].

Both the β and ε phases have hexagonal lattice structures with very similar lattice constants (a = 4.01 Å for both phases; c = 16.64 and 16.70 Å for the β and ε phases, respectively) and contain two quadruple layers per unit cell [20]. However, their crystal symmetries differ because of their distinct stacking sequences. In β-InSe, adjacent layers are translated by one-third of the primitive lattice vector and rotated by 60° relative to one another. This AB′ stacking sequence results in a centrosymmetric *P*6_3_/*mmc* ($D_{6h}^{4}$) space group. By contrast, the ε phase has a parallel AB stacking sequence without the 60° interlayer twist; this configuration breaks inversion symmetry and results in a non-centrosymmetric *P*$\bar{6}$*m*2 ($D_{3h}^{1}$) space group. Unlike the hexagonal β and ε phases, the γ phase adopts a rhombohedral crystal structure characterized by an ABC stacking order. In this configuration, adjacent layers are translated in plane without any twist. This extended repeating sequence comprises three quadruple layers per unit cell and yields an OOP lattice parameter of approximately 24.95 Å. The γ phase belongs to the non-centrosymmetric *R*3*m* ($C_{3v}^{5}$) space group. This lack of inversion symmetry enables unique symmetry-driven physical phenomena, including SHG and sliding ferroelectricity, both of which are being investigated for next-generation memory and optoelectronic applications [16,21].

First-principles calculations indicated that the energy differences among various stacking configurations can be smaller than the thermal energy under typical vapor-phase growth conditions [20]. Consequently, synthesized multilayer InSe often exhibits a high density of stacking faults or nanoscale intermixing of different phase domains. This structural heterogeneity induces spatial fluctuations in electronic band alignments that can strongly influence charge transport and exciton dynamics in the material.

### 2.2. Electronic-Band-Structure Evolution

#### 2.2.1. Small Effective Electron Mass and High Thermal Velocity

**Figure 2:**
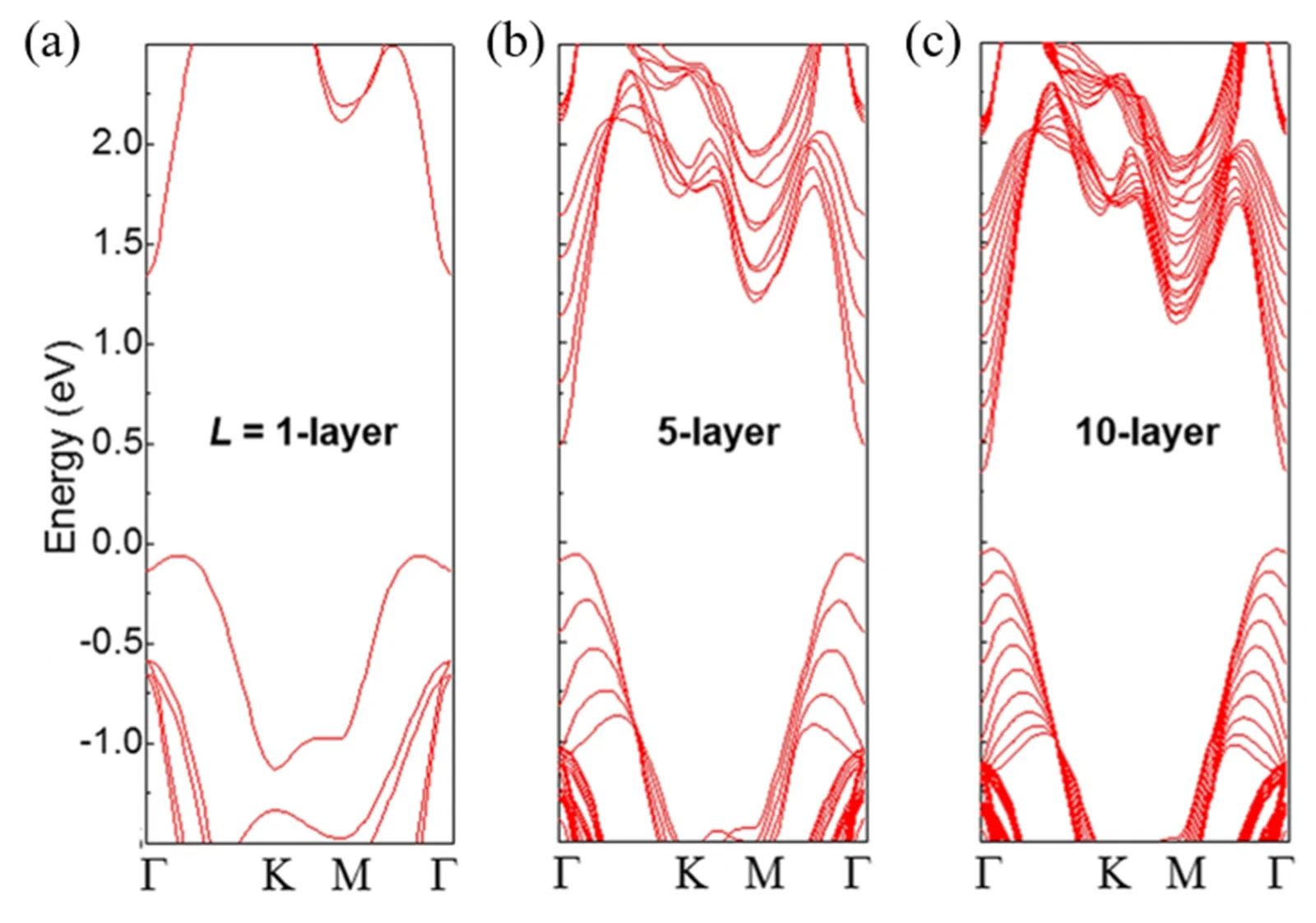
(**a**–**c**) Calculated band structures of (**a**) one-, (**b**) five-, and (**c**) 10-layer InSe. Reproduced with permission [11].

The asymmetric electron and hole transport in layered InSe arises from substantial differences in the curvature of the conduction and valence bands near band edges. The valence band maximum (VBM) exhibits relatively weak dispersion, yielding a large hole effective mass that often exceeds 0.60 *m*_0_ (Figure 2) [11]. By contrast, the conduction band minimum (CBM) at the Γ point exhibits a parabolic shape with strong dispersion and high curvature. First-principles many-body calculations have yielded IP and OOP electron effective masses of 0.12 *m*_0_ and 0.09 *m*_0_, respectively, for γ-InSe [5]. These values are substantially lower than those reported for conventional 2D TMDs (e.g., approximately 0.45 *m*_0_ for MoS_2_) and 3D Si (0.19 *m*_0_) [7]. According to the relationship $v_{th}\propto 1/\sqrt{m_{e}^{*}}$, the small $m_{e}^{*}$ of InSe results in an electron *v*_th_ exceeding 1.3 × 10^7^ cm s^−1^ at room temperature. This value exceeds those of conventional Si (1.2 × 10^7^ cm s^−1^) and widely studied 2D TMDs (typically <0.8 × 10^7^ cm s^−1^).

When a transistor channel is aggressively scaled to the sub-10 nm regime, charge transport transitions from a scattering-dominated diffusive regime to a quasi-ballistic or purely ballistic regime. In this quantum limit, the injection velocity of charge carriers from the source is fundamentally limited by the intrinsic *v*_th_ of the channel material. Consequently, the superior *v*_th_ of electrons in InSe enables them to traverse ultrashort channels with minimal transit delay and negligible phonon scattering. A recent study of ballistic InSe transistors demonstrated the advantage of InSe over Si and typical 2D semiconductors [7]. This characteristic positions InSe as the leading channel candidate for future high-speed, low-power integrated circuits.

#### 2.2.2. Thickness-Dependent Direct-to-Indirect Bandgap Crossover

The band structure of InSe evolves with thickness in a manner opposite to that of typical semiconducting TMDs. Bulk β- and γ-InSe are direct-bandgap semiconductors with band edges at the Γ point of the Brillouin zone and reported bandgap energies of approximately 1.28 and 1.29 eV, respectively [22]. As thickness decreases, the CBM remains centered at Γ, whereas the VBM gradually shifts away from Γ, resulting in indirect-bandgap characteristics [11]. In a previous study, density-functional calculations established this behavior by demonstrating that the upper valence band evolves into an inverted Mexican-hat profile as the number of layers decreases (Figure 2). Recently, researchers directly visualized this crossover using spatially resolved angle-resolved photoemission spectroscopy, observing an indirect bandgap in monolayer and bilayer InSe and a direct bandgap in InSe with six or more layers [23].

First-principles studies have reported that this behavior arises from the distinct orbital compositions of the band edges [24]. The conduction band edge of multilayer InSe is derived from In s orbitals and Se s and p_z_ orbitals and remains fixed at the Γ point regardless of thickness. By contrast, competition between bands with different orbital characteristics determines the valence band edge. In the bulk limit, the short In–Se bond length enhances the OOP overlap between the p_z_ orbitals of In and Se, raising the p_z_-derived valence band at Γ to the highest valence-band energy and yielding a direct gap. As InSe is thinned, the In–Se bond length increases and OOP p_z_–p_z_ coupling weakens, lowering the energy of the Γ-point p_z_ band. Consequently, the p_x_/p_y_-dominated band, which peaks at the Q point, emerges as the VBM. The VBM therefore shifts from Γ to Q, creating a momentum mismatch with the CBM at Γ and rendering the bandgap indirect. Table 1 summarizes the structural and electronic properties of various InSe polytypes. 

### 2.3. Emerging Ferroelectricity

Unlike conventional perovskite oxides subject to severe finite-size effects and depolarization fields at the nanoscale, layered InSe exhibits robust OOP and IP spontaneous polarizations down to atomically thin dimensions [15]. However, the existence, orientation, and reversibility of these polarizations depend sensitively on the polytype, number of layers, and degree of stacking disorder.

The earliest experimental evidence of ferroelectric switching in InSe was reported in thin β-InSe nanoflakes, with room-temperature piezoelectric response measurements revealing hysteretic OOP and IP loops [27]. Although the exact mechanism of ferroelectricity remains unresolved, these results are unusual because β-InSe has a centrosymmetric *P*6_3_/*mmc* structure and is traditionally considered non-ferroelectric. Remarkably, recent studies have demonstrated that the macroscopic symmetry of β-InSe can be artificially broken [15]. Under sufficiently strong vertical electric fields, the covalently bonded Se–In–In–Se quadruple layers can undergo relative IP sliding along the armchair direction, transforming the lattice into 1T-InSe. This field-driven atomic displacement produces interlinked IP and OOP ferroelectric responses.

**Figure 3:**
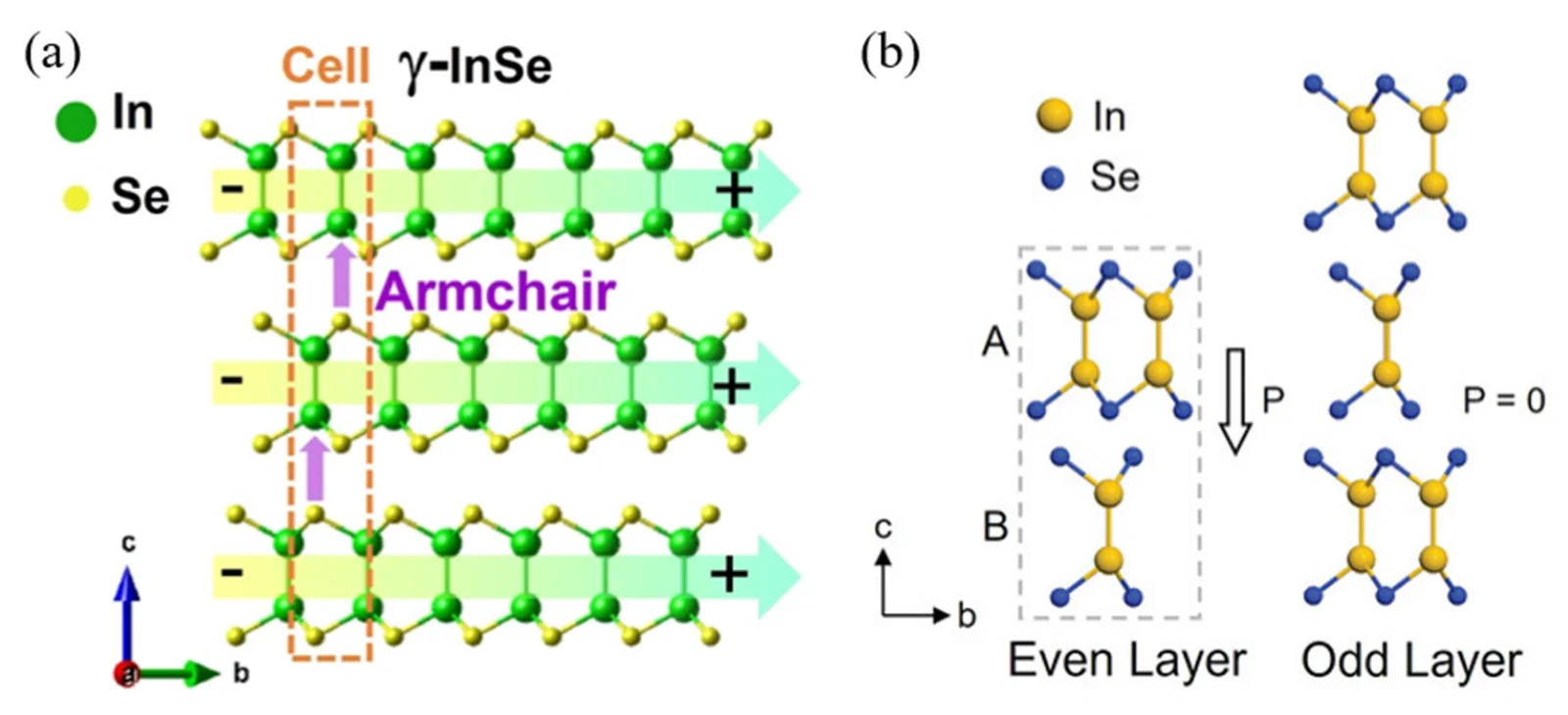
(**a**) Atomic configuration of γ-InSe, illustrating OOP polarization along the OOP direction and IP polarization along the armchair axis (indicated by arrows in the middle panel). Green and yellow spheres represent In and Se atoms, respectively. Reproduced with permission [16]. (**b**) Side views of the crystal structures of bilayer (left) and tri-layer (right) ε-InSe. Net OOP polarization occurs only in even-layer ε-InSe. Yellow and blue spheres represent In and Se atoms, respectively. Reproduced with permission [17].

Unlike centrosymmetric β-InSe, γ-InSe inherently exhibits both IP and OOP polarization because of its ABC stacking sequence (Figure 3a) [16]. However, conventionally grown γ-InSe has a high stacking-fault density that severely disrupts long-range polar order and weakens spontaneous polarization. To address this structural limitation, Sui et al. have added a trace amount of yttrium (Y) to improve crystal quality. Notably, interstitial Y atoms stabilize the γ-InSe structure, eliminate planar stacking faults, and simultaneously induce a subtle rhombohedral distortion. This deformation involves intralayer compression along the c-axis, particularly a reduction in the vertical distance between Se atoms, together with continuous interlayer pre-sliding. This synergistic atomic reconstruction not only widens the optical bandgap but also enhances the net vertical dipole moment. Consequently, Y-doped γ-InSe exhibits an exceptionally high effective piezoelectric constant with values of approximately 4.0 and 14.1 pm V^−1^ for 120 and 14 nm-thick flakes, respectively.

ε-InSe displays another form of structure-dependent polarization, which exhibits a strict odd–even dependence on the number of layers (Figure 3b) [17]. In its even-layer form (e.g., a bilayer), ε-InSe has an AB stacking configuration and belongs to the C_3v_ point group. This atomic arrangement inherently breaks both horizontal mirror symmetry and twofold rotational symmetry, inducing net charge transfer between adjacent layers and producing robust OOP polarization. By contrast, adding a third layer changes the stacking sequence to ABA. In this odd-layer case, the structure belongs to the centrosymmetric D_3h_ point group, and the vertical dipole moments associated with the two adjacent *vdW* gaps cancel, resulting in zero net OOP polarization.

## 3. Synthesis and Wafer-Scale Preparation of 2D InSe

### 3.1. Top-Down Exfoliation Strategies

#### 3.1.1. Mechanical Exfoliation

**Figure 4:**
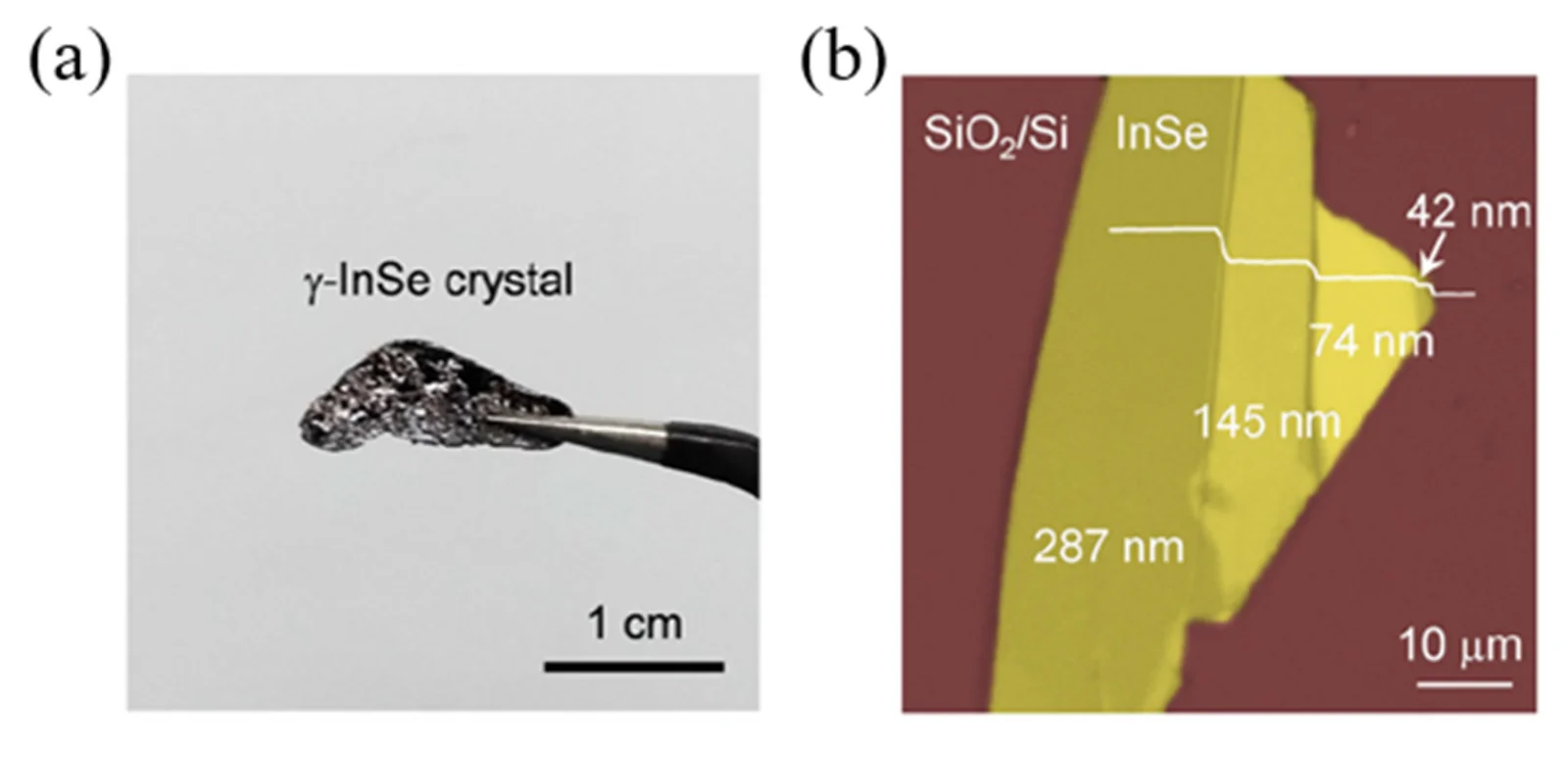
(**a**) Representative photograph of a bulk γ-InSe crystal grown by the Bridgman method. (**b**) Optical image of a mechanically exfoliated γ-InSe flake on an SiO_2_/Si substrate. The solid white line represents the height profile of the γ-InSe flake. Reproduced with permission [30].

Mechanical exfoliation remains a straightforward method for isolating *vdW* 2D materials, enabling an investigation of their intrinsic properties. In early studies of atomically thin InSe, bulk γ-InSe single crystals, typically synthesized via the Bridgman method, were cleaved with adhesive tape and transferred onto insulating substrates such as SiO_2_/Si or mica [28,29]. Owing to the weak OOP *vdW* interactions, cleavage yields thin flakes with atomically flat terraces and well-defined quadruple-layer steps, thereby enabling investigations of thickness-dependent optical properties (Figure 4) [30]. The principal advantage of mechanical exfoliation is the quality of the isolated flakes. Because the method involves no wet-chemical intercalation, sonication, or energetic particle bombardment, exfoliated InSe generally preserves its single-crystalline lattice and native basal plane. Consequently, most research on 2D InSe has relied on mechanically exfoliated flakes to explore its fundamental characteristics.

Despite these advantages, tape-assisted exfoliation is inherently probabilistic. Flake thickness, lateral dimensions, and spatial location cannot be predetermined and must instead be identified after transfer, limiting reproducibility and throughput. This process typically yields flakes with areas below 1000 μm^2^. The resulting lack of spatial scalability and low throughput make mechanical exfoliation infeasible for the wafer-scale integration of commercial electronic devices. Consequently, although mechanical exfoliation remains an essential tool for proof-of-concept devices, these severe limitations have prompted research into advanced liquid-phase processes and scalable bottom-up vapor deposition strategies.

#### 3.1.2. Liquid-Phase and Electrochemical Exfoliation

**Figure 5:**
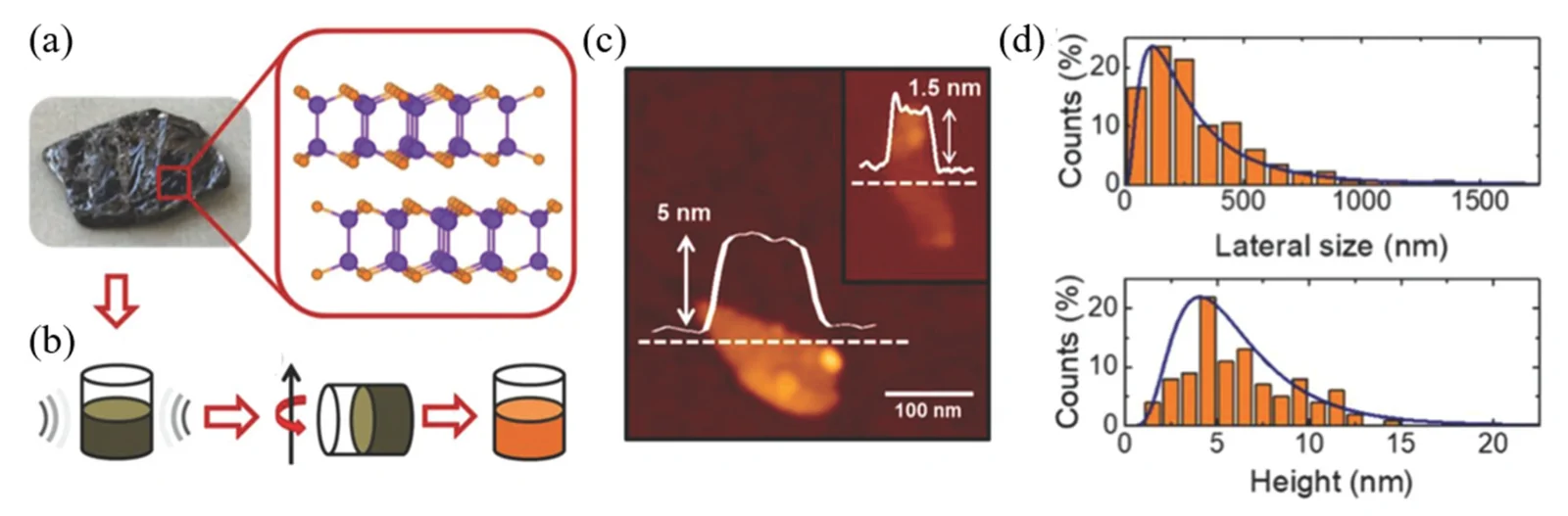
(**a**) Photograph (left) and crystal structure (right) of a bulk β-InSe crystal. (**b**) Schematic of the LPE process, showing the sonication (left) and ultracentrifugation (middle) stages and the final stable dispersion of exfoliated β-InSe flakes (right). (**c**) Atomic force microscopy (AFM) image of an exfoliated InSe flake. The solid white lines indicate height profiles along the dashed white lines. The inset presents an AFM image of an exfoliated InSe flake with a thickness of 1.5 nm. (**d**) Statistical distributions of the lateral sizes (top) and thicknesses (bottom) of dispersed InSe flakes. Reproduced with permission [34].

To address the severe spatial scalability and throughput limitations of mechanical exfoliation, liquid-phase exfoliation (LPE) has been employed for the mass production of 2D InSe. Conventional LPE processes often use high-boiling-point organic solvents, such as N-methyl-2-pyrrolidone and dimethylformamide, or surfactant-containing aqueous solutions to stabilize the exfoliated nanosheets [31,32,33]. However, removing residual solvent layers or insulating surfactant layers requires heat treatment, which inevitably damages InSe. Petroni et al. demonstrated surfactant-free exfoliation of β-InSe in low-boiling-point alcohol media (Figure 5) [34]. In 2-propanol, stable dispersions with concentrations up to 0.11 g L^−1^ were obtained, and the resulting flakes had lateral dimensions ranging from tens of nanometers to several micrometers and thicknesses of approximately 1–20 nm. Importantly, no detectable conversion to In_2_Se_3_ or oxide phases occurred. These results confirm the applicability of direct exfoliation using a surfactant-free solvent for InSe but suggest that post-exfoliation separation remains necessary to obtain a thin, uniform film due to the wide thickness distribution.

Because of its high surface area-to-volume ratio, exfoliated InSe is highly sensitive to dissolved oxygen in aqueous solutions, leading to rapid chemical decomposition and the formation of irreversible In–O–Se trap states [35,36]. To mitigate this degradation and oxidation, a deoxygenated, surfactant-free cosolvent strategy has recently been proposed [37]. A key advantage of this strategy is its use of a binary mixture of ethanol and water, both of which have low boiling points. When implemented in a sealed, argon-purged sonication environment, this cosolvent approach strongly inhibits the formation of InO_x_ and SeO_x_ defects.

Electrochemical molecular intercalation has emerged as a highly effective, solution-processable approach for exfoliating InSe into monolayers [15,38]. In a typical electrochemical exfoliation process, a bulk InSe crystal serves as the cathode in an electrolyte containing tetraethylammonium bromide (TEAB) dissolved in dimethylformamide [15]. When a negative bias is applied to InSe, organic cations such as tetrahexylammonium (THA^+^) intercalate into the vdW gap according to the following reaction: InSe + *x*THA^+^ + *x*e^−^ → (THA^+^)*x*InSe^x−^. This aggressive intercalation weakens interlayer adhesion, induces OOP volume expansion, and ultimately causes delamination into high-purity single- or few-layer nanosheets. He et al. recently modified this approach to improve the quality and stability of exfoliated InSe [38]. In that study, tetraheptylammonium bromide (THAB)-based intercalation and exfoliation were performed in an air-free environment using nitrogen-degassed solvents. When the same process was conducted in air, the resulting InSe was oxidized and damaged, leading to poor electrical switching behavior. By contrast, under oxygen- and moisture-controlled conditions, the exfoliated nanosheets remained pure InSe, exhibited a narrow thickness distribution with monolayer purity above 98%, and could be assembled into 4-in. thin films with a roughness of approximately 2 nm.

### 3.2. Bottom-Up Thin-Film Growth Techniques

#### 3.2.1. Chemical Vapor Deposition (CVD) and Physical Vapor Transport

To overcome the limitations of conventional top-down exfoliation, including small lateral dimensions and probabilistic production of flakes, vapor-phase deposition techniques have been used to synthesize large-scale 2D InSe suitable for practical device applications. Unlike conventional TMDs, which have relatively wide stability windows, InSe exists in several stoichiometric compositions, including InSe, In_2_Se_3_, In_4_Se_3_, and In_6_Se_7_, each with a narrow stability window [39]. Consequently, small deviations in the local Se/In ratio drive growth toward In_2_Se_3_ or other In–Se phases rather than the targeted InSe phase.

**Figure 6:**
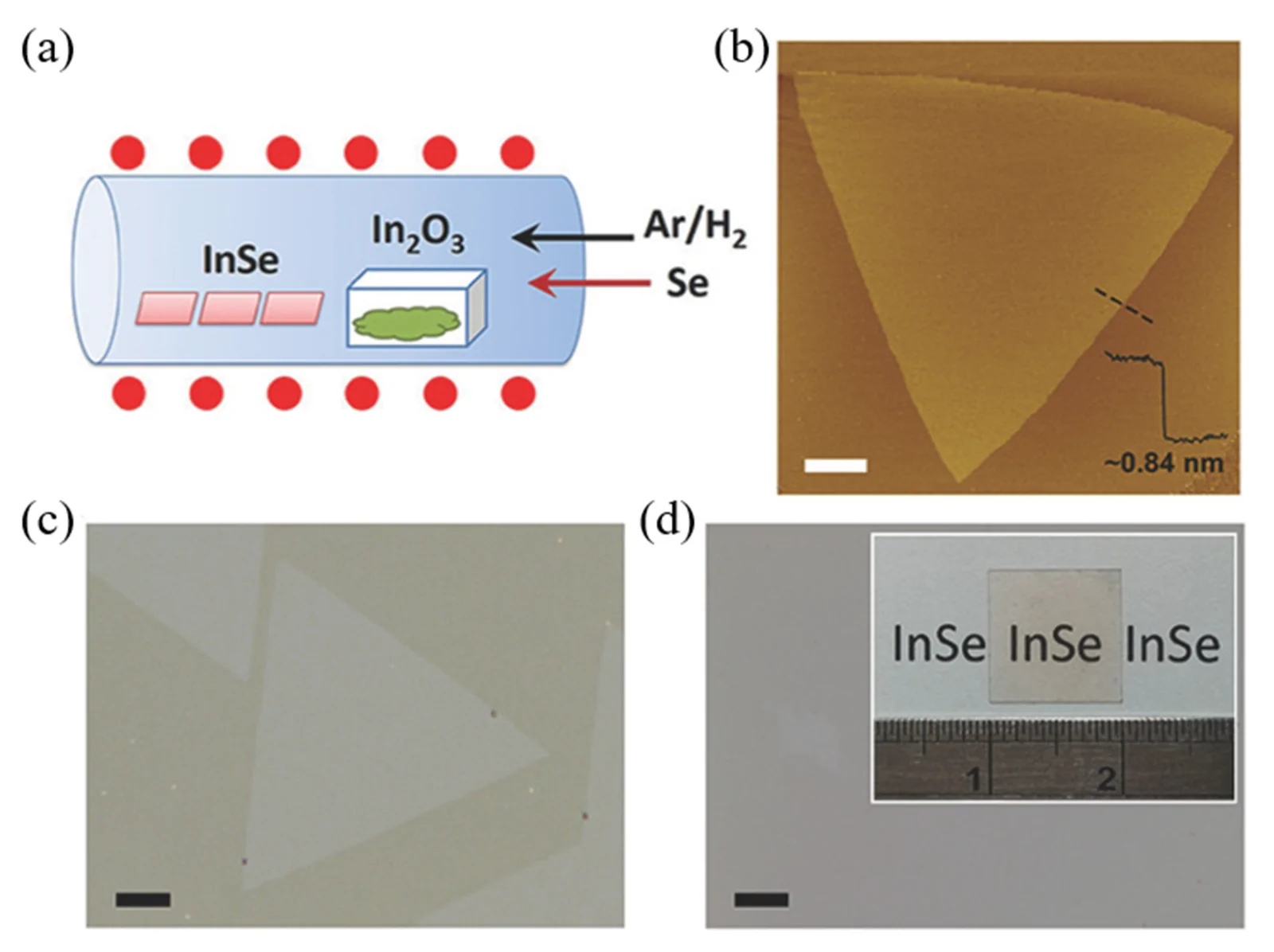
(**a**) Schematic of the CVD setup for InSe growth. (**b**) AFM topographic image of an InSe monolayer flake. The solid black line represents the height profile measured along the dashed black line, corresponding to a thickness of 0.84 nm. (**c**,**d**) Optical images of InSe monolayer flakes (**c**) and a continuous film (**d**). The inset in (**d**) presents a photograph of a centimeter-scale InSe monolayer film synthesized on a mica substrate. The scale bars are 10 mm. Reproduced with permission [40].

Chang et al. demonstrated the synthesis of monolayer InSe films using a powder-based CVD process (Figure 6) [40]. In this study, indium oxide (In_2_O_3_) and Se powders served as the In and Se sources, respectively, while an H_2_/Ar gas mixture acted as the carrier gas. InSe synthesized on a mica substrate exhibited high crystallinity and phase purity, with no other allotropes present, and spanned an area exceeding 1 × 1 cm^2^. Another research group explained the basis of this phase-pure InSe growth [14]. In that study, InSe grew under similar conditions, except that low-melting-point indium iodide (InI) was used as the In source. Selective growth of the InSe phase over the undesired In_2_Se_3_ phase was governed primarily by two tunable parameters: the hydrogen concentration in the carrier-gas flow and the Se supply, which was regulated by the Se sublimation temperature. In the absence of H_2_ or at low H_2_ concentrations, only In_2_Se_3_ formed. As the H_2_ concentration gradually increased, a phase transition occurred, increasing InSe formation and eventually suppressing In_2_Se_3_ formation. Reducing the Se supply produced a similar effect. These H_2_- and Se-dependent effects are attributed to the combined influences of a hydrogen-assisted phase transition and Se deficiency. Hydrogen first provides the activation energy required to initiate In_2_Se_3_ formation, after which surface-adsorbed H_2_ lowers the energy barrier between the two phases, facilitating the conversion of In_2_Se_3_ to InSe. Simultaneously, a Se-deficient environment can induce the formation of the InSe phase. Because excessive H_2_ induces etching and a low Se supply yields dendritic morphologies, a pure InSe phase can be obtained only within a narrow window defined by the synergistic effect of a high H_2_ concentration and a limited Se supply.

Beyond controlling precursor flux or Se supply to suppress competing stoichiometries, growth temperature can also be modulated to change the resulting InSe phase. Balakrishnan et al. demonstrated that a spatial temperature gradient within the furnace provides a similarly effective route for the phase engineering of InSe [41]. By sublimating Bridgman-grown γ-InSe powder at 600 °C and positioning substrates at locations with temperatures between 500 and 580 °C, they selectively synthesized γ-InSe and α-, β-, and γ-In_2_Se_3_ in a single process. Hu et al. reported another chemical vapor transport process in which InSe and NH_4_Cl powders were first converted into volatile InCl and H_2_Se species at 600 °C [42]. The low substrate temperature induced the reverse reaction, producing β-InSe and α-In_2_Se_3_ at 400 and 450 °C, respectively.

#### 3.2.2. Metal–Organic CVD (MOCVD)

Although conventional powder-based vapor deposition methods have successfully produced 2D materials, their reliance on solid-source sublimation hinders precise control of precursor evaporation rates. Such control is particularly important for InSe because the growth of InSe, In_2_Se_3_, and other In–Se compounds is highly sensitive to subtle changes in the Se/In ratio. Although MOCVD does not completely resolve the phase-selectivity issue, it offers a viable approach by enabling more precise control of precursor supply than conventional transport growth methods. 

**Figure 7:**
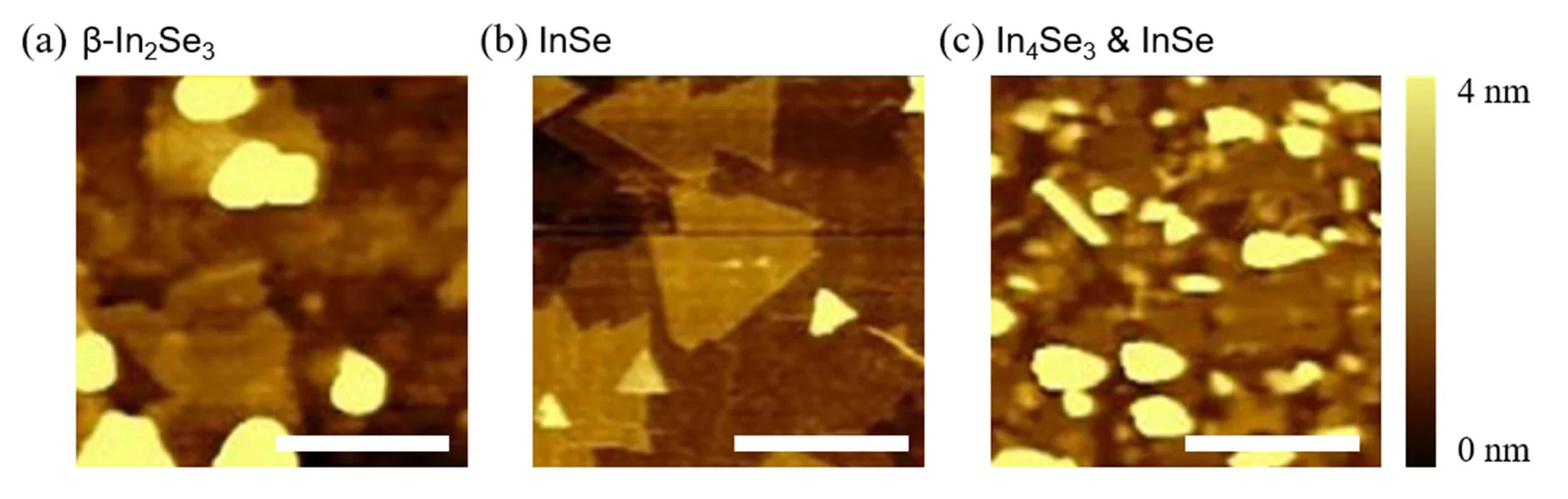
(**a**–**c**) AFM topographic images of representative In_x_Se_y_ samples grown at (**a**) 450, (**b**) 400, and (**c**) 350 °C. The scale bars are 500 nm. Reproduced with permission [44].

Song et al. reported the selective growth of 2D InSe and In_2_Se_3_ on a 2-in. c-plane sapphire substrate using trimethylindium (TMIn) and dimethyl selenide (DMSe) in a vertical-showerhead MOCVD reactor [43]. In this process, the growth of InSe or In_2_Se_3_ is governed not simply by the magnitude of the Se flux but by the period of pulsed DMSe delivery. Under constant DMSe flow, the InSe growth window remained narrow, frequently leading to transformation into In_2_Se_3_ or the formation of In-rich droplets. By contrast, pulsed DMSe delivery broadened the InSe growth window; promoted lateral coalescence; and enabled wafer-scale, layer-by-layer growth down to the monolayer limit. Because efficient thermal decomposition of DMSe requires temperatures exceeding 400–500 °C, growth was conducted predominantly at 500 °C. However, this high thermal budget is incompatible with back-end-of-line (BEOL) integration, which requires process temperatures below 400 °C. Recently, phase-pure InSe films were also synthesized on c-plane sapphire in a horizontal MOCVD reactor using TMIn and diisopropyl selenide (DiPSe), which has a lower activation energy for thermal decomposition than DMSe (Figure 7) [44]. By optimizing the DiPSe/TMIn ratio, the authors synthesized phase-pure InSe at 400 °C, whereas deviations from this optimal temperature led to secondary In_x_Se_y_ formation.

#### 3.2.3. Pulsed Laser Deposition (PLD) and Molecular Beam Epitaxy (MBE)

**Figure 8:**
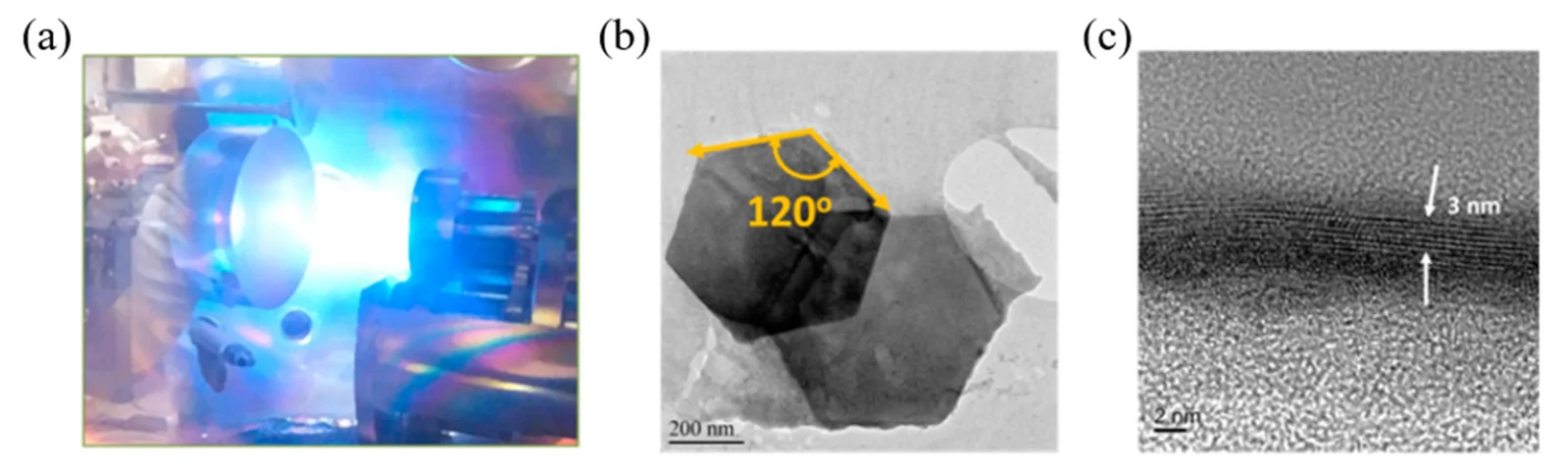
(**a**) Photograph of the laser ablation process. (**b**) Low-magnification transmission electron microscopy (TEM) image of PLD-grown InSe. (**c**) Cross-sectional TEM image of a tri-layer InSe film. Reproduced with permission [25].

PLD and MBE are alternative bottom-up methods using elemental or solid sources for thin-film deposition rather than relying on gaseous precursor reactions. Under high- or ultrahigh-vacuum conditions, these methods effectively eliminate complex chemical reactions and the need for carrier-gas injection typical of CVD, instead relying on direct sublimation or ablation of solid-source materials. Specifically, PLD enables material transfer to the substrate while preserving target stoichiometry. High-energy laser pulses eject gas-phase ions and atoms, allowing precise control of thin-film thickness by adjusting the number of laser pulses (Figure 8) [25]. However, increasing the substrate temperature during deposition inevitably causes severe Se deficiency in the resulting films because Se is highly volatile relative to In. To address this issue, Bergeron et al. separated deposition from crystallization [39]. In this strategy, an amorphous InSe thin film was deposited at room temperature to preserve PLD target stoichiometry and subsequently crystallized through post-deposition thermal annealing. The subsequent phase evolution depended strongly on the annealing temperature because of the complexity of the In–Se phase diagram. At relatively low annealing temperatures above 220 °C, the initial amorphous film underwent partial crystallization, resulting in the coexistence of InSe and In_4_Se_3_ phases. As the annealing temperature increased further into the optimal 325–425 °C window, a phase-pure, stable, crystalline InSe film was obtained. Consequently, precise temperature control during post-deposition thermal annealing is imperative for guiding phase evolution and controlling phase transitions to obtain phase-pure, high-quality InSe thin films.

MBE offers distinct advantages for InSe synthesis by enabling independent and precise control of the In and Se fluxes [45,46]. Hilse et al. demonstrated MBE-based growth of InSe films on GaAs(111)B substrates [20]. Because β-, γ-, and ε-InSe have highly comparable formation energies, the resulting films typically exhibit mixed nanoscale polytype domains. In another study, solid-phase epitaxy was used for phase engineering [47]. In this process, a crystalline α-In_2_Se_3_ film was subsequently exposed to a high In flux in the absence of Se, resulting in the thermodynamic transformation of the entire film into pure γ-InSe. This phase transition was driven by the high diffusivity of In and the excellent IP lattice matching between α-In_2_Se_3_ and γ-InSe.

The structural characteristics, transport properties, and BEOL compatibility of InSe obtained across different synthesis strategies are summarized in Table 2.

## 4. Next-Generation Device Applications

### 4.1. High-Performance FETs

#### 4.1.1. Ballistic Transport and Sub-3 nm-Node Logic Devices

**Figure 9:**
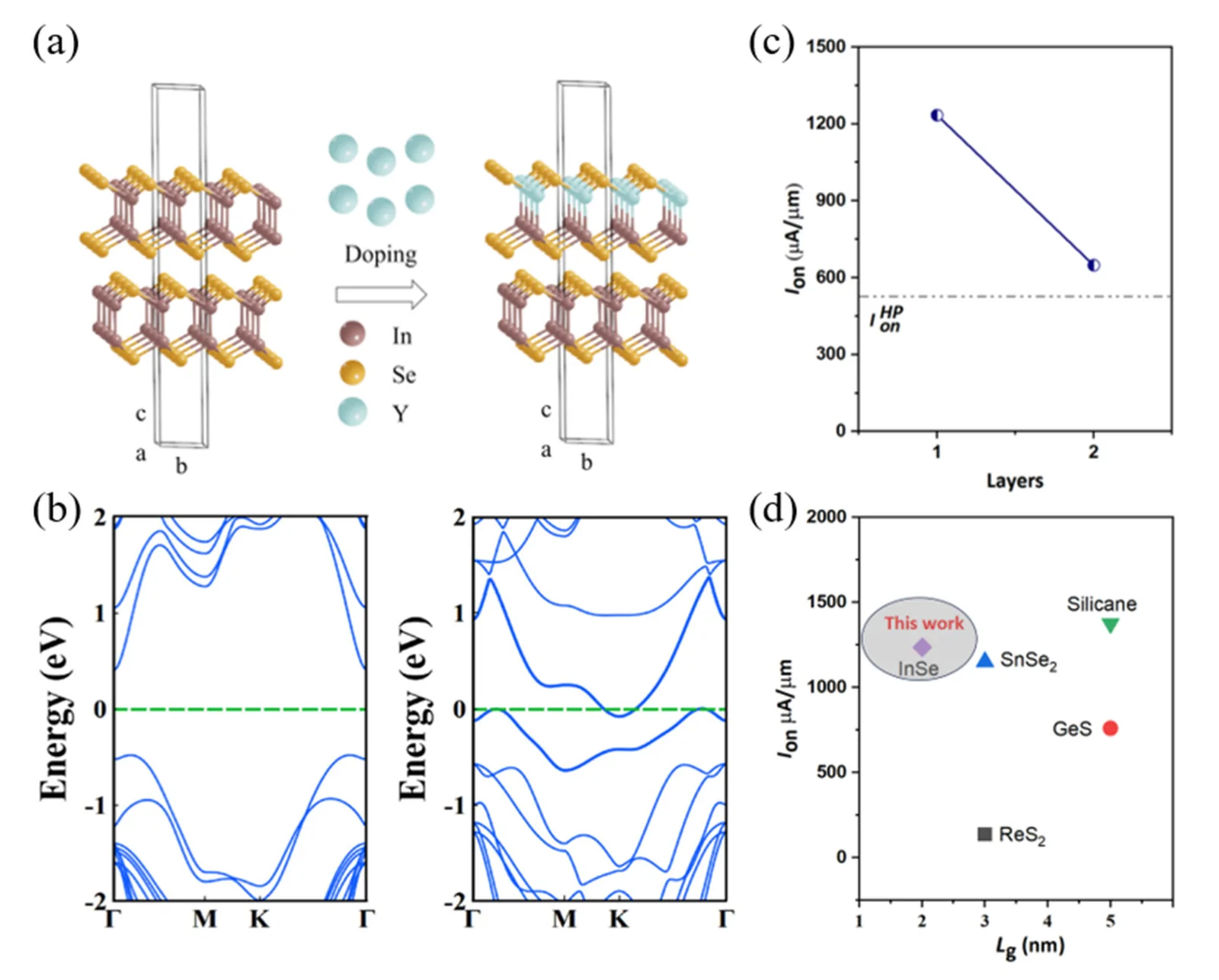
(**a**,**b**) Crystal (**a**) and band (**b**) structures of intrinsic (left) and Y-doped (right) bilayer InSe. Reproduced with permission [48]. (**c**) Calculated *I*_on_ values for monolayer and bilayer β-InSe FETs with a gate length (*L*_g_) of 2 nm. The dashed line indicates the high-performance International Technology Roadmap for Semiconductors (ITRS) device requirement. (**d**) Benchmarking of Ion for previously reported transistors based on monolayer (ML) 2D materials [49,50,51,52,53]. Reproduced with permission [49].

Recent advances have positioned InSe as a highly promising channel material for ultra-scaled ballistic transistors because of its fundamental physical advantages over Si and 2D TMDs [7]. Specifically, its smaller $m_{e}^{*}$ enables a higher electron *v*_th_. To realize these theoretical benefits, a double-gate FET with a 10 nm channel length was fabricated using tri-layer InSe and 2.6 nm-thick HfO_2_ as the channel and gate dielectric, respectively. Furthermore, doping the top InSe layer with Y atoms transformed the semiconducting contact region into a semi-metallic Y–InSe phase, thereby establishing an ideal ohmic contact at the metal–InSe interface (Figure 9) [48]. This contact-engineering approach yielded a low contact resistance of 62 Ω μm. Consequently, the InSe FET exhibited a near-ideal room-temperature ballistic ratio of 83% in the saturation region. The devices also exhibited outstanding performance, including a transconductance of 6 mS μm^−1^ and an on-state current exceeding 1 mA μm^−1^ at an ultralow supply voltage of 0.5 V. The dual-gate structure ensured excellent electrostatic control and effectively suppressed short-channel effects, with a subthreshold swing of 75 mV/decade and drain-induced barrier lowering of 22 mV V^−1^. Ultimately, these exceptional ballistic transport characteristics of InSe FETs arise from the synergistic combination of intrinsically high carrier injection velocity and Y-doped ohmic contacts. Table 3 summarizes the electrical performance metrics of short-channel InSe FETs in comparison with state-of-the-art TMD-based FETs.

Beyond the experimental success of 10 nm FETs using tri-layer InSe, recent theoretical research has also identified the ultimate scaling limit of this promising material (Figure 9) [49]. For instance, using ab initio quantum transport simulations, researchers investigated the electrical performance of ML and bilayer (BL) β-InSe transistors scaled to ultrashort gate lengths of 2 and 3 nm. Notably, at a gate length of 2 nm, the ML and BL β-InSe transistors were predicted to achieve outstanding on-state currents of 1236 and 648 μA μm^−1^, respectively, both exceeding the ITRS target of 528 μA μm^−1^. The ML InSe transistor also exhibited superior gate electrostatics and a lower subthreshold swing than its BL counterpart. These performance differences are attributed primarily to increased interlayer interactions and reduced electrostatic gate control in thicker multilayer structures. These simulations indicate that appropriately engineered, atomically thin β-InSe channels can maintain robust ballistic transport and excellent switching capabilities even at sub-3 nm physical gate lengths. 

#### 4.1.2. Mobility Enhancement via Dielectric Engineering and Interface Passivation

**Figure 10:**
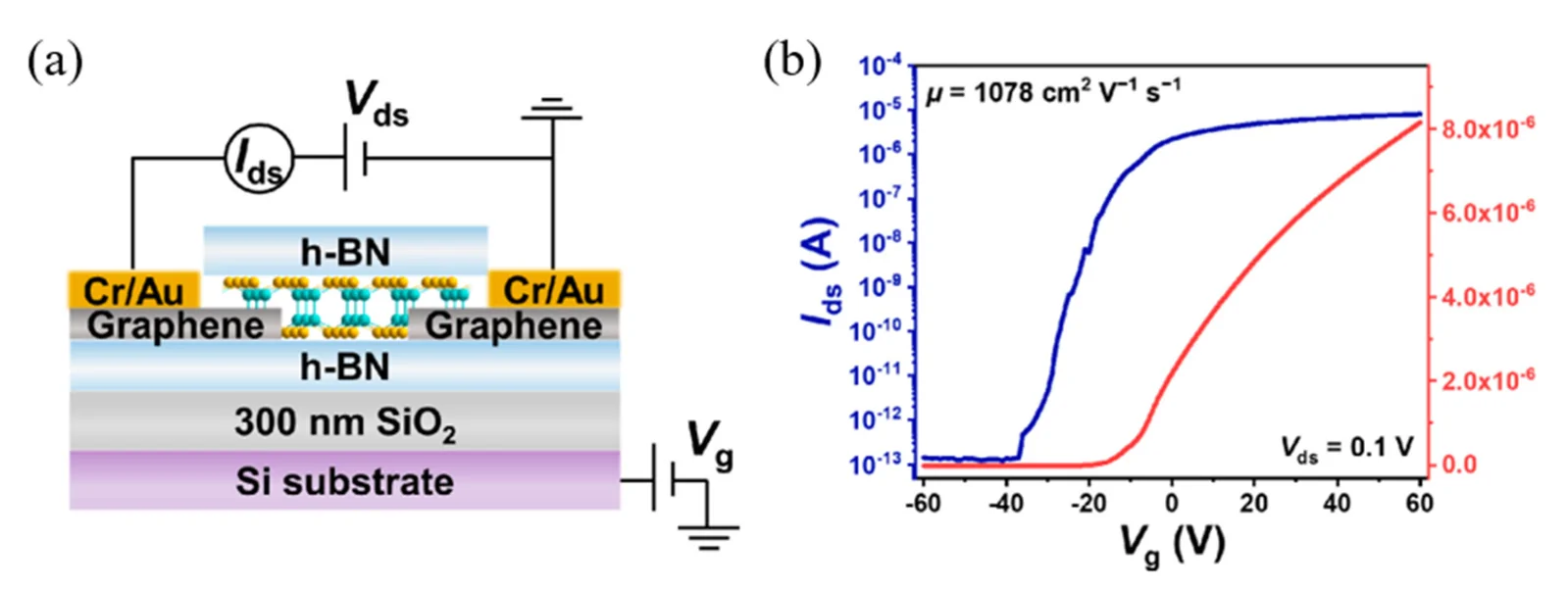
(**a**) Schematic of an *h*-BN-encapsulated InSe FET incorporating an *h*-BN/SiO_2_ back-gate insulator and graphene electrodes. (**b**) Transfer characteristics of the encapsulated InSe FET at *V*_ds_ = 0.1 V on logarithmic (blue line) and linear (red line) scales. Reproduced with permission [63].

Although InSe theoretically has a small conduction-band effective mass, carrier mobility in practical FETs is rarely determined by intrinsic band structure alone. In these systems, the dielectric environment also strongly affects carrier transport because the channel is exposed to oxide-related charged centers, surface adsorbates, and polar vibrational modes. At interfaces with conventional SiO_2_ or directly deposited high-κ oxides, hydroxyl terminations, residual moisture, and interfacial trap states introduce long-range Coulomb disorder, and polar dielectric surfaces additionally induce remote phonon scattering. Consequently, the measured field-effect mobility of InSe can fall below the transport limit determined by its small electron effective mass. Early gate-dielectric engineering strategies have effectively addressed these interfacial issues. For instance, in a previous study, InSe on a poly(methyl methacrylate) (PMMA)/Al_2_O_3_ substrate exhibited a room-temperature electron mobility of 1055 cm^2^ V^−1^ s^−1^ and a subthreshold swing of 300 mV/decade, considerably outperforming InSe on a bare Al_2_O_3_ substrate, which exhibited corresponding values of 64 cm^2^ V^−1^ s^−1^ and 600 mV/decade, respectively [61]. In a similar study, Sucharitakul et al. reported that PMMA-supported InSe devices consistently outperformed devices fabricated on bare SiO_2_ or Si_3_N_4_ substrates [62]. Alternatively, *vdW* dielectrics can isolate the InSe channel from underlying oxide substrates. Among these materials, hexagonal boron nitride (*h*-BN) provides an atomically flat, dangling-bond-free surface that effectively suppresses surface roughness-induced carrier scattering. Consequently, recently developed *vdW*-integrated InSe transistors using *h*-BN as both an encapsulation layer and a gate dielectric achieved room-temperature electron mobilities above 10^3^ cm^2^ V^−1^ s^−1^, on/off ratios of approximately 10^8^, and negligible electrical degradation after one month in air (Figure 10) [63].

#### 4.1.3. Steep-Slope Operation Beyond the Thermionic Limit

Conventional FETs are fundamentally constrained by the room-temperature thermionic limit, which limits the SS to a minimum of 60 mV/dec. Alternatively, TFET configurations enable sub-thermionic transport via band-to-band tunneling (BTBT). Miao et al. demonstrated a heterojunction tunnel triode by integrating a few-layer n-type γ-InSe flake onto degenerately p-doped Si. Using a vertically stacked Ti/Au top gate with an AlO_x_ dielectric layer, this device efficiently modulates the energy band alignment across the *vdW* interface [64]. Given the atomic-scale thickness of the InSe nanoflake and the heavily doped Si substrate, top-gate modulation occurs almost entirely within the 2D InSe layer, altering the interfacial potential. As a result, a small gate-voltage swing is required to align the valence band of Si with the conduction band of InSe, thereby facilitating efficient BTBT transport. Remarkably, these heterojunction devices achieved an extremely steep minimum *SS* of 6.4 mV dec^−1^, maintaining sub-60 mV dec^−1^ behavior across four decades of drain current with an average *SS* of 34.0 mV dec^−1^. In contrast, control FETs fabricated using identical InSe flakes exhibited a minimum *SS* of 100 mV dec^−1^, suggesting that the sub-thermionic switching originates from the *vdW* heterojunction. Furthermore, an exceptionally small electron effective mass ($m_{e}^{*}$ ≈ 0.14 *m*_0_) enhances the BTBT probability.

### 4.2. Nonvolatile Memory and Neuromorphic Computing

The nonvolatile operational behavior of InSe-based devices relies on multiple physical origins, which can be categorized into three major operational mechanisms. First, sliding ferroelectricity, arising from the non-centrosymmetric crystal symmetry of γ-InSe, provides intrinsic nonvolatile switching. The second involves charge storage in a floating gate or dielectric charge-trapping layer, where trapped carriers screen the external gate field to modulate the threshold voltage. The third relies on defect states originating from the underlying dielectric, adsorbed atmospheric molecules, or self-limiting native oxidation on the InSe surface.

#### 4.2.1. Transistors Based on Sliding Ferroelectricity

**Figure 11:**
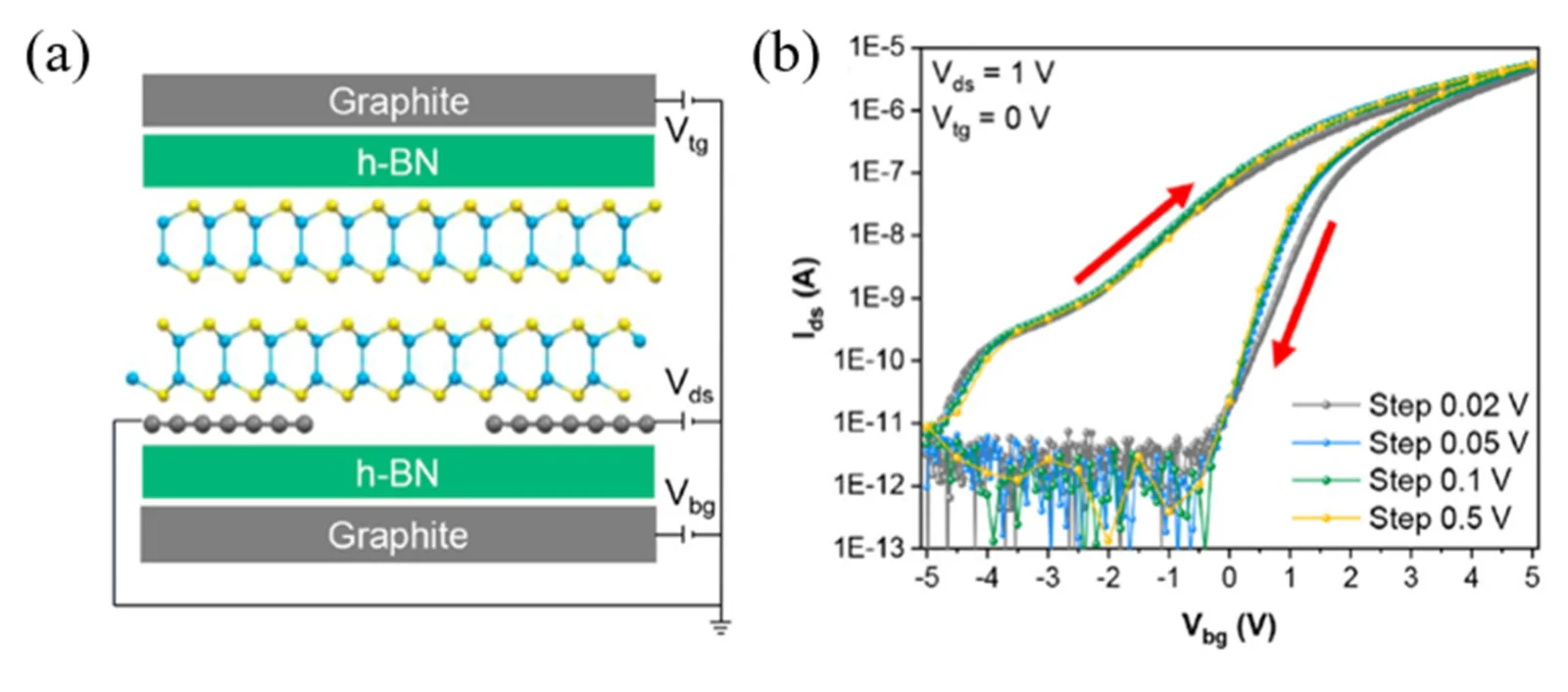
(**a**) Schematic of a ferroelectric-channel transistor wherein γ-InSe, graphene, and *h*-BN serve as the ferroelectric channel, electrodes, and dielectric layers, respectively. (**b**) Transfer characteristics of the InSe ferroelectric-channel FET at *V*_ds_ = 1 V and *V*_tg_ = 0 V with different *V*_bg_ steps ranging from 0.02 to 0.5 V. Reproduced with permission [65].

Beyond conventional volatile logic applications, the unique sliding ferroelectricity of 2D InSe provides a promising basis for next-generation nonvolatile memory and neuromorphic computing. In γ-InSe, polarization switching is associated with changes in the lateral alignment between adjacent Se–In–In–Se quadruple layers across the *vdW* gap rather than with the ion off-centering mechanism that dominates conventional oxide ferroelectrics. In principle, this intrinsic structural configuration suppresses defect-mediated ion migration, a process that typically limits the endurance of conventional ferroelectric memories. In early transistor architectures, γ-InSe was employed directly as the semiconducting channel, unlike the conventional ferroelectric FET (FeFET) configuration, wherein the ferroelectric material serves exclusively as the gate dielectric (Figure 11) [65]. In this structure, γ-InSe and graphene serve as the active ferroelectric channel and contact electrode, respectively. The device operates through polarization-controlled carrier redistribution that induces Schottky barrier variations at the contact interfaces. This differs from the standard ferroelectric-gate modulation used in conventional FeFET stacks. The resulting device achieved a memory window of 4.5 V and an on/off ratio of 10^6^. An alternative InSe-based FeFET configuration was demonstrated by integrating γ-InSe as the ferroelectric top-gate layer and *h*-BN as the insulating spacer on a MoS_2_ channel [66]. In this configuration, the γ-InSe layer does not serve as the active readout channel; instead, its switchable polarization electrostatically controls the underlying MoS_2_ channel, thereby modulating its conductance. This device exhibited a 6.8 V memory window within a ±6 V sweep range and a conductance modulation exceeding 10^4^. Furthermore, it demonstrated robust reliability, with switching endurance beyond 10^3^ cycles and a projected retention time on the order of 10 years.

#### 4.2.2. Charge-Trapping and Floating-Gate Memories

Alternatively, by introducing a charge-trapping layer or a floating gate, non-volatility can be achieved while maintaining 2D InSe as the high-mobility conduction channel. In representative *vdW* floating-gate transistors, the 2D InSe channel is decoupled from the charge-trapping floating gate by an atomically flat *h*-BN tunneling barrier [67]. Upon applying a sufficient gate bias, charge carriers tunnel across the ultrathin *h*-BN barrier and become trapped in the floating gate. In particular, the atomically sharp and pristine interfaces in InSe/*h*-BN/graphene heterostructures allow ultrafast carrier transport into and out of the graphene layer within 160 ns. Furthermore, nonvolatile trapping does not require metallic or semi-metallic layers. For instance, oxygen-plasma treatment of *h*-BN introduces a high density of localized trap states capable of capturing electrons just as effectively as a graphene floating gate. Wang et al. demonstrated an InSe/*h*-BN/O_2_-*h*-BN *vdW* heterostructure, in which a defective, plasma-treated O_2_-*h*-BN layer functions as a tunable charge-trapping medium and the underlying pristine *h*-BN provides an atomically flat tunneling barrier. [68]. Consequently, the trapped charges effectively screen the gate electric field, producing a wide nonvolatile threshold-voltage shift of up to 93 V. Similarly, directly integrating the defective trapping layer adjacent to the channel yields a broad memory window of approximately 130 V [69]. 

#### 4.2.3. Synaptic Devices for Artificial Neural Networks

The traditional von Neumann architecture faces severe limitations in handling complex, data-intensive tasks because the physical separation of its processing and memory units causes substantial energy consumption [70,71]. To overcome these limitations, researchers have actively developed brain-inspired neuromorphic computing systems that mimic the highly parallel, fault-tolerant, and ultralow-power operation of biological neural networks [72,73,74]. Consequently, artificial synaptic devices that emulate biological signal transmission and memory functions have emerged as candidates for next-generation information technology.

**Figure 12:**
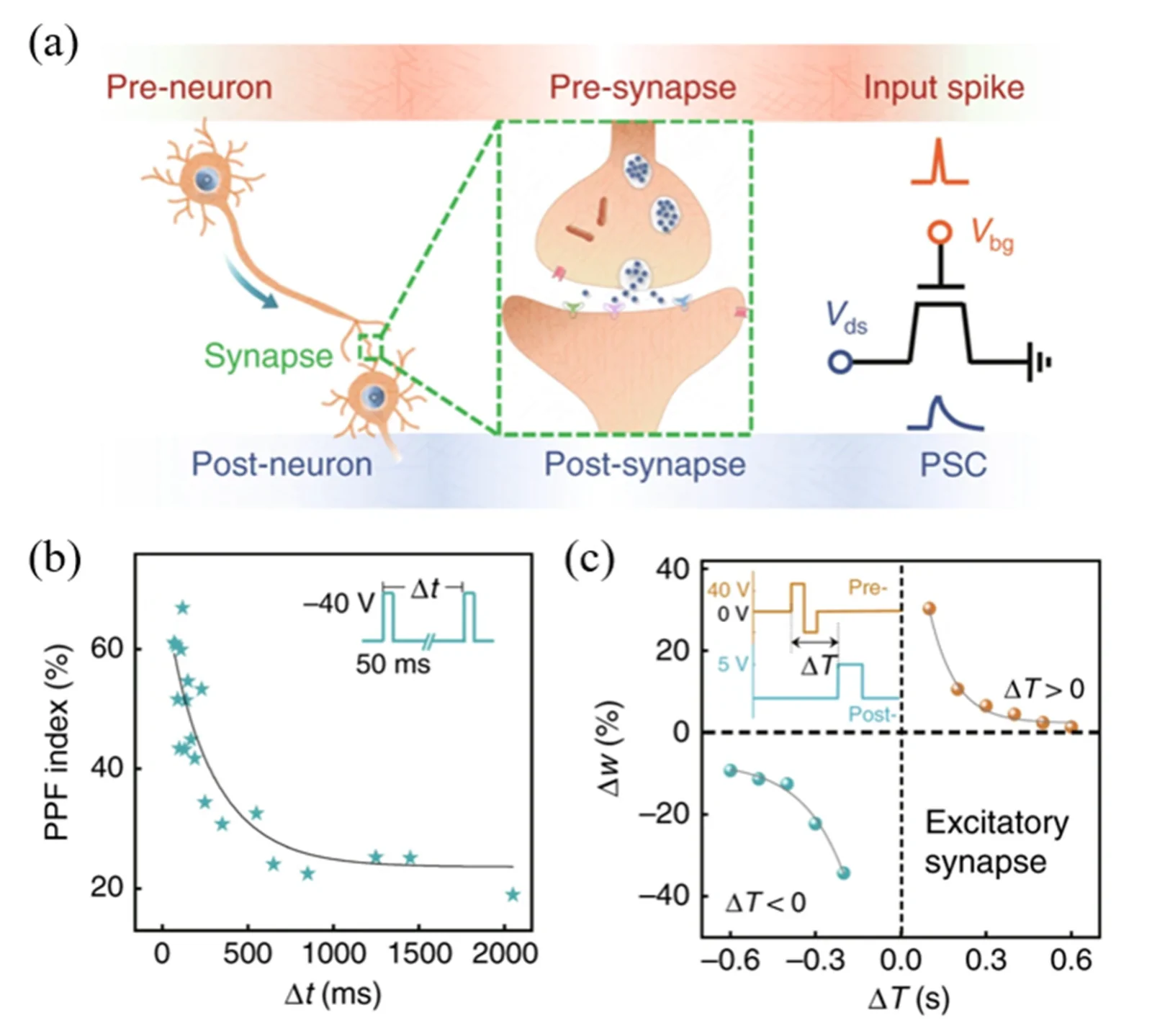
(**a**) Schematic of a biological synapse and the InSe-based artificial synaptic device. (**b**) Extracted PPF index as a function of spike time interval (Δ*t*); the PPF index is defined as (*A*_2_ − *A*_1_)/*A*_1_, where *A*_1_ and *A*_2_ correspond to the first and second postsynaptic current (PSC) peak amplitudes, respectively. The solid line represents a double-exponential fit to the experimental data. (**c**) Synaptic weight updates (Δ*w*) as a function of relative spike timing (Δ*T*), emulating STDP characteristics, with exponential fits. Δw is calculated as (ΔPSC_2_ − ΔPSC_1_)/ΔPSC_1_, where ΔPSC_1_ and ΔPSC_2_ are the changes in PSC after presynaptic and postsynaptic spikes, respectively. Reproduced with permission [79].

Among the various materials explored for synaptic emulation, 2D *vdW* semiconductors have emerged as promising candidates because of their atomic-scale thickness, high switching speeds, and excellent charge-transport properties [75,76,77,78]. In particular, InSe has garnered attention for constructing artificial synapses because it exhibits an exceptionally high intrinsic carrier mobility of 1000 cm^2^ V^−1^ s^−1^ [62]. This high intrinsic mobility increases device response speed while reducing operational power consumption. Although InSe is highly sensitive to ambient conditions and undergoes electrical degradation through native oxidation, this inherent environmental sensitivity has been recently exploited to enable advanced neuromorphic functions [79]. The InSe FETs comprised an InSe channel on an SiO_2_/Si substrate, and exposure to ambient conditions naturally induced the formation of an ultrathin, amorphous InO_x_ layer at the channel–dielectric interface. This interfacial native oxide functioned as an efficient charge-trapping medium rather than as a source of electrical degradation, thereby enabling controllable modulation of synaptic weight. Depending on the applied gate voltages, electrons were dynamically trapped in or released from the InO_x_ layer during programming or erasing, respectively, enabling deterministic and precise modulation of channel conductance. Consequently, these InSe-based FETs exhibited nonvolatile memory performance, including stable multilevel storage and robust retention exceeding 2000 s. Furthermore, the device successfully emulated essential forms of biological synaptic plasticity, such as paired-pulse facilitation (PPF) and spike-timing-dependent plasticity (STDP) (Figure 12). Building on these advances in three-terminal InSe-based FETs, researchers recently demonstrated unprecedented energy efficiency in two-terminal InSe devices [80]. By tuning the applied pulse frequencies and amplitudes, the fabricated device exhibited exceptionally low energy consumption of only 1.1 fJ per spike. Table 4 summarizes the key operational characteristics and switching performance of InSe-based memory and neuromorphic devices.

## 5. Remaining Challenges and Future Outlook

Despite advances in material synthesis, several bottlenecks hinder the integration of 2D InSe into manufacturable electronic devices. Specifically, achieving spatial and stoichiometric uniformity across a large-area substrate remains the main challenge because local variations in thickness and composition substantially reduce device yield and reliability. Although recent breakthroughs in MOCVD and PLD have enabled the production of continuous wafer-scale films, these films remain primarily polycrystalline. Furthermore, the nearly identical thermodynamic formation energies of the β, γ, and ε phases lead to the local coexistence of polytypes. The resulting extensive grain boundaries and structural heterogeneity provide centers for severe carrier scattering and cause spatial fluctuations in the bandgap, respectively, thereby inducing device-to-device performance variations across the wafer.

The second unresolved issue concerns compatibility with integration processes. Although *vdW* semiconductors relax the lattice-matching constraints of conventional epitaxy, large-area InSe growth still requires a specific crystallographic template. This requirement severely limits the direct monolithic integration of InSe onto amorphous oxide dielectrics or industrial BEOL substrates. Consequently, researchers rely heavily on transfer processes that inevitably introduce mechanical wrinkles, polymer residues, and interfacial traps, ultimately degrading device performance. 

Environmental instability constitutes the third bottleneck. Freshly synthesized or exfoliated InSe layers undergo rapid ambient oxidation, which severely degrades their intrinsic electronic properties. Although ideal InSe has a dangling-bond-free basal plane, exfoliated or vapor-deposited InSe surfaces remain highly susceptible to atmospheric degradation. This degradation generally begins with the rapid physisorption of ambient oxygen and moisture molecules at intrinsic surface defects, such as Se vacancies. This initial physisorption is quickly followed by irreversible chemisorption, which spontaneously generates amorphous InO_x_ and SeO_x_ species. These native oxides induce interfacial trap states and suppress charge-carrier transport. Consequently, high-quality ultrathin InSe devices are commonly assembled under an inert atmosphere and immediately passivated after exfoliation, most often by *h*-BN encapsulation or oxide barriers deposited via atomic layer deposition. Advanced encapsulation and passivation strategies are required to overcome this environmental degradation and achieve compatibility with Si complementary metal–oxide–semiconductor process flows. Overall, 2D InSe has evolved from a model system for studying fundamental physical phenomena into an engineering challenge for commercialization.

## Figures and Tables

**Table 1:** Structural and electronic properties of different InSe polytypes.

Polytype	Stacking Sequence	Inversion Symmetry	Space Group	Bandgap (eV)	Refs.
β-InSe	AB′	Centrosymmetric	*P*6_3_/*mmc*	1.26 (bulk) to 1.93 (2L)	[15,20]
ε-InSe	AB	Non-centrosymmetric	$P\bar{6}$*m*2	1.26 (bulk) to 2.20 (1L)	[20,25]
γ-InSe	ABC	Non-centrosymmetric	*R*3*m*	1.25 (bulk) to 1.69 (3L)	[20,26]

**Table 2:** Comparison of structural, electrical, and BEOL-compatibility characteristics of 2D InSe synthesized via various top-down and bottom-up routes.

Preparation Method	Substrate	Precursor/Source	Substrate Temperature (°C)	Lateral Size	Thickness (nm)	Polytype	Mobility (cm^2^ V^−1^ s^−1^)	BEOL Compatibility	Refs.
ME	SiO_2_/Si	Bulk InSe crystal	RT	N/A	2.4	N/A	N/A	No (not scalable)	[7]
ME	SiO_2_/Si	Bulk γ-InSe crystal	RT	Tens of μm	5.8–287	γ-InSe	N/A	No (not scalable)	[30]
ME	SiO_2_/Si	Bulk γ-InSe crystal	RT	Tens of μm	4–75	γ-InSe	N/A	No (not scalable)	[28]
LPE	SiO_2_/Si	Bulk InSe crystal	RT	4-inch	0.8	N/A	90–120	Yes	[38]
LPE	Al_2_O_3_	Bulk InSe crystal	RT	1.5 cm	41	N/A	19	Yes	[37]
LPE	SiO_2_/Si	Bulk InSe crystal	RT	A few μm	1.4	β-InSe	N/A	Yes	[15]
LPE	SiO_2_/Si	Bulk β-InSe crystal	RT	A few μm	1–20	β-InSe	N/A	Yes	[34]
CVD	Mica	In_2_O_3_ and Se powders	630	1 cm	0.84	ε-InSe	30	No (not scalable)	[40]
CVD	Mica	InI and Se powders	600	Tens of μm	3–12.5	ε-InSe	N/A	No (not scalable)	[14]
CVT	Mica	InSe and NH_4_Cl powders	400–450	10 μm	4–15	β-InSe, α-Inn_2_Se_3_	10.8	No (not scalable)	[42]
PVT	ε-GaSe	Bulk γ-InSe crystal	500–580	Hundreds of μm	2.8–4	γ-InSe and α-, β-, and γ-In_2_Se_3_	N/A	No (not scalable)	[41]
PLD	SiO_2_/Si	Bulk InSe target	600	1 cm	0.8–20	ε-InSe	10–70	No (high temperature)	[25]
PLD and post-annealing	SiO_2_/Si	InSe and In_2_Se_3_ targets	Deposition: RT, Annealing: 400	1 cm	4.5–23.4	ε-InSe	0.2	Yes	[39]
MOCVD	Al_2_O_3_	TMIn and DiPSe	350–400	Wafer-scale	0.8–4.1	γ-InSe	N/A	Yes	[44]
MOCVD	Al_2_O_3_	TMIn and DMSe	500	2-inch	0.8	N/A	0.04	Yes	[43]
MOCVD	Al_2_O_3_	TMIn and DMSe	500	2-inch	5.9	N/A	4.5	Yes	[43]
MBE	Si(111)	In and Se	275–375	3-inch	3.7–30	γ-InSe	N/A	Yes	[45]
MBE	GaAs(111)B	In and Se	350	Wafer-scale	40	γ-, β-, and ε-InSe	N/A	Yes	[20]
MBE	Glass, Al_2_O_3_, Al foil	In and Se	550	2-inch	22	γ-InSe	N/A	No (high temperature)	[46]
MBE and post-annealing	Al_2_O_3_	In and Se	Deposition: 560, Annealing: 740	Wafer-scale	N/A	γ-InSe		No (high temperature)	[47]

Abbreviations: TMIn, trimethylindium; DiPSe, diisopropyl selenide; DMSe, dimethyl selenide; RT, room temperature.

**Table 3:** Comparison of key device metrics for short-channel 2D FETs based on InSe and TMDCs.

Material	Layer	Gate Length (nm)	Contact	Dielectric	Mobility (cm^2^ V^−1^ s^−1^)	Contact Resistance (Ω μm)	*SS* (mV dec^−1^)	*I*_on_ (μA μm^−1^) /*V*_DS_ (V)	*I*_on_/*I*_off_	Refs.
InSe	3L	10	Y/Ti/Au	HfO_2_ (2.6 nm)	N/A	62	75	1.20 × 10^3^/0.5	10^7^	[7]
InSe	3L	20	Y/Ti/Au	HfO_2_ (2.6 nm)	N/A	62	62	1.35 × 10^3^/0.5	10^8^	[7]
InSe	3L	10	Y/Ti/Au	HfO_2_ (2.6 nm)	N/A	N/A	79	1.00 × 10^3^/0.5	10^7^	[54]
InSe	3L	480	Y/Ti/Au	HfO_2_ (2.6 nm)	287	N/A	67	8.92 × 10^2^/1.2	10^6^	[54]
MoS_2_	1L	35	Bi/Au	SiN_x_ (100 nm)	22	123	N/A	1.14 × 10^3^/1.5	10^6^	[55]
MoS_2_	1L	20	Sb/Au	HfO_2_ (14 nm)	N/A	42	185	1.23 × 10^3^/1.0	10^8^	[56]
MoS_2_	2L	1	Ni/Au	ZrO_2_ (5.8 nm)	N/A	N/A	65	1.00 × 10^1^/1.0	10^6^	[57]
MoS_2_	1L	7.5	1T-MoS_2_	HfO_2_ (10 nm)	N/A	N/A	120	2.50 × 10^2^/1.0	10^7^	[58]
MoS_2_	1L	14	Ni/Au	HfO_2_ (16 nm)	N/A	2300	87	N/A	10^6^	[59]
MoS_2_	2L	40	Bi/Au	Al_2_O_3_ (25 nm)	N/A	300	N/A	1.20 × 10^3^/2.0	N/A	[60]
WS_2_	1L	120	Bi/Au	SiN_x_ (100 nm)	19	N/A	N/A	3.50 × 10^2^/1.5	10^7^	[55]

**Table 4:** Performance summary of InSe-based non-volatile memory and neuromorphic devices.

Architecture Design	Channel Material	Gate Dielectric	Pulse Voltage Amplitude (V)	Pulse Width (ms)	Retention (s)	Endurance (Cycles)	Neuromorphic Task Demonstration	Refs.
Ferroelectric channel transistor	InSe	*h*-BN	±25	N/A	1.0 × 10^3^	N/A	N/A	[65]
Ferroelectric FET	MoS_2_	γ-InSe/*h*-BN	±6	1.0 × 10^–3^	1.0 × 10^3^	1.0 × 10^3^	N/A	[66]
Ferroelectric channel transistor	InSe	N/A	±2	N/A	1.0 × 10^3^	N/A	N/A	[66]
Charge-trapping transistor	InSe	InO_x_/SiO_2_	±80	5.0 × 10^1^ to 5.0 × 10^2^	2.0 × 10^3^	1.0 × 10^2^	MNIST digit recognition	[79]
Two-terminal memristor	InSe	N/A	1 to 6	1.0 × 10^–3^	N/A	2.4 × 10^3^	Image edge detection	[80]
Charge-trapping transistor	InSe	O_2_-*h*-BN/*h*-BN	−80 to −120	1.0 × 10^2^ to 1.0 × 10^3^	N/A	N/A	N/A	[69]
Floating-gate-like FET	InSe	*h*-BN/O_2_-*h*-BN	−30 to −80	1.0 × 10^1^ to 2.5 × 10^2^	2.0 × 10^2^	1.0 × 10^1^	N/A	[68]

## Data Availability

No new data were created or analyzed in this study. Data sharing is not applicable to this article.
